# Pollution, Ecological Risk and Source Identification of Heavy Metals in Sediments from the Huafei River in the Eastern Suburbs of Kaifeng, China

**DOI:** 10.3390/ijerph191811259

**Published:** 2022-09-07

**Authors:** Bingyan Jin, Jinling Wang, Wei Lou, Liren Wang, Jinlong Xu, Yanfang Pan, Jianbiao Peng, Dexin Liu

**Affiliations:** 1 College of Geography and Environmental Science, Henan University, Kaifeng 475004, China; 2 Key Laboratory of Geospatial Technology for the Middle and Lower Yellow River Regions, Ministry of Education, Henan University, Kaifeng 475004, China; 3 School of Environment, Henan Normal University, Xinxiang 453007, China; 4 Henan Overseas Expertise Introduction Center for Discipline Innovation (Ecological Protection and Rural Revitalization along the Yellow River), Kaifeng 475004, China

**Keywords:** heavy metal, pollution characteristics, ecological risk, sources identification, river sediments

## Abstract

Rivers in urban environments are significant components of their ecosystems but remain under threat of pollution from unchecked discharges of industrial sewage and domestic wastewater. Such river pollution, particularly over the longer term involving heavy metals, is an issue of worldwide concern regarding risks to the ecological environment and human health. In this study, we investigate the long-term pollution characteristics of the Huafei River, an important urban river in Kaifeng, China. River sedimentary samples were analyzed, assessing the degree and ecological risk of heavy metal pollution using the geo-accumulation index and potential ecological risk index methods, whilst Pearson’s correlation, principal component and cluster analyses were used to identify the sources of pollution. The results show that heavy metal concentrations are significantly higher than their corresponding fluvo-aquic soil background values in China, and the geo-accumulation indexes indicate that of the eight heavy metals identified, Hg is most prevalent, followed in sequence by Cd > Zn > Cu > Pb > Ni > As > Cr. The potential ecological risk index of the Huafei River is very high, with the potential ecological risk intensity highest in the midstream and downstream sections, where it is recommended that pollution control is carried out, especially concerning Hg and Cd. Long-term sequence analysis indicates that Cu and Pb dropped sharply from 1998 to 2017, but rebounded in 2019, and that Zn shows a continuous decreasing trend. Four main sources for the heavy metal contaminants were identified: Cr, Cu, Ni, Pb, Zn and Hg derived mainly from industrial activities, traffic sources and natural sources; Cd originated mainly from industrial and agricultural activities; whilst As was mainly associated with industrial activities. Thus, special attention should be paid to Hg and Cd, and measures must be taken to prevent further anthropogenic influence on heavy metal pollution.

## 1. Introduction

It is generally accepted that, in urban environments, the prevalence of rivers are not only mere features of the landscape, but, in some instances, they are also of significant cultural importance; furthermore, they also form important components of urban ecosystems. Rivers provide a range of economic and social benefits, including flood control and drainage, water supplies and sources of recreation, to name but a few; moreover, as living ecosystems in their own right, urban rivers are of significance in the context of the wider ecological environment. However, urban rivers are under threat, especially from the acceleration of urbanization, which has a propensity for large amounts of industrial sewage and domestic wastewater to be discharged into them, resulting in the river becoming polluted, particularly concerning heavy metal pollution of river sediment [1,2]. At the junction of the liquid–solid two-phase interface, river sediment is an important part of the water environment. It is a reservoir for various pollutants in the water [3,4] which, in effect, records changes in environmental information over time. Moreover, it is also an important carrier for studying the accumulation of heavy metals and persistent organic pollutants [2,5], as well as being a medium for reviewing the ecological risks to the urban water environment [5,6,7].

Heavy metals, with their immutable characteristics of non-biodegradability, toxicity and bio-accumulation, have a negative impact on the health of humans, as well as ecosystems [8,9,10] and, therefore, their occurrence in rivers needs to be strictly controlled. Consequently, this conclusion essentially stimulated further research activity into heavy metal pollution in river sediments which includes recent studies on the temporal and spatial distribution [11,12], source identification [13,14], pollution assessment [5] and ecological restoration [15] of heavy metals. Although these studies, and others, have made progress, they are generally lacking in long-term research on both heavy metal accumulation and ecological risk. Currently, Hakanson’s potential ecological risk index method [16] is predominantly used in ecological risk assessments, although, in order to use it correctly in assessments, the potential ecological risk coefficient ($E_{r}$) and potential ecological risk index (*RI*) grading standards must be adjusted according to the specific types and quantities of pollutants involved in the assessment [17]. However, existing studies mostly follow Hakanson’s grading standard directly, that is, without any adjustments to ($E_{r}$) and (*RI*) [18,19], thus affecting the reliability of the evaluation results. Nevertheless, despite established practice, our study included all necessary adjustments.

The Huafei River rises in northeast Kaifeng; its water sources are mainly from the Yellow River, but include industrial wastewater and domestic sewage, etc. Thus, it is an important river in terms of the urban landscape, flood discharge and sewage discharge. Moreover, there is a large area of cultivated land in the east of the Huafei River, so it is also an important river for agricultural irrigation but has had a history of sewage pollution for some 58 years. Irrigated areas, which have been polluted by the Huafei River for a long time, have seen their soils subjected to the continuous accumulation of heavy metals, significantly threatening the soil, ecological environment and, consequently, human health [14,20,21]. Since the end of the 20th century, several studies have focused on the Huafei River and the associated areas irrigated by sewage, and has carried out a series of studies on heavy metal pollution, including heavy metal pollution of the soil–crop system in the areas irrigated by sewage [22], the responses of local fauna to heavy metal pollution in the areas irrigated by sewage [23], heavy metal pollution in soil of the areas irrigated by sewage [24] and heavy metal pollution of the sediment in the Huafei River [15,25], etc. The objectives of this paper were: (1) to collect sediment samples from the Huafei River to determine and analyze their physical and chemical properties {pH, organic matter (OM), total nitrogen (TN) and total phosphorus (TP)} and the prevalence of eight heavy metals (As, Cd, Cr, Cu, Hg, Ni, Pb and Zn); (2) to evaluate the degree of contamination and assess the ecological risks in the river by calculating the geo-accumulation index and potential ecological risk index, recalling the data on heavy metals in research over the last 20 years; (3) to identify the possible sources of these heavy metals based on Pearson’s correlation analysis, principal component analysis and cluster analysis. It is anticipated that the findings of this study will provide theoretical references for the effective control and scientific management of heavy metal pollution in the Huafei River and its surrounding areas.

## 2. Materials and Methods

### 2.1. Study Area

The study area in the Kaifeng basin is in the central-eastern region of Henan province, China, on the northeast to southwest sloping plain area, which is midstream and downstream of the Yellow River. The region is characterized by a temperate monsoon climate, with an average annual temperature of 14 °C and precipitation of 627.5 mm, which is mainly concentrated in the period between July to August. The parent material of the soil in Kaifeng is alluvium from the Yellow River, and the main soil type is fluvo-aquic soil [21]. Within the study area, the Huafei River was selected for investigation because it is subject to industrial wastewater discharges from a range of sources, including the Kaifeng fertilizer plant, paper factory, instrument manufacturer, pharmaceutical factory, zinc smelting plant and the Kaifeng carbon enterprise [25]. Also known as Dongjiao Furrow, the Huafei River flows from north to south into the Huiji River, which is a tributary of the Huaihe River, in the southeast of Kaifeng City. The Huafei River is approximately 15 km long with an average velocity of 0.4 m·s^−1^, an average depth of 0.67 m, a width of 3.98 m, a bed gradient of one in 4000 and a basin area of about 30 km^2^.

### 2.2. Sample Collection and Analysis

**Figure 1:**
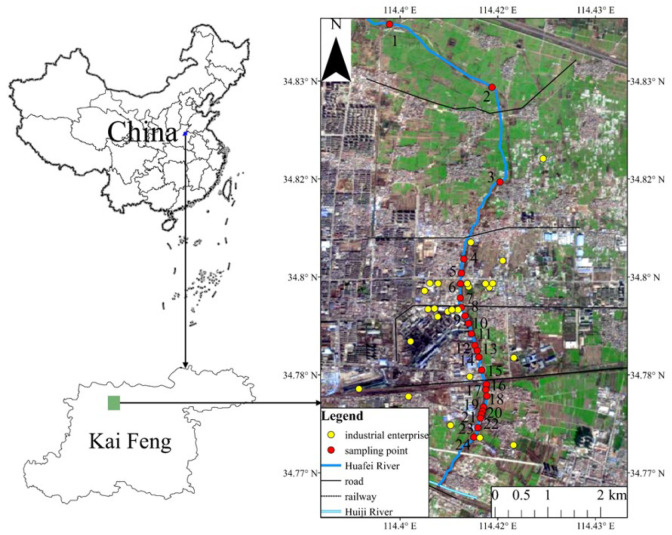
Sampling location map of the study area.

A total of 24 surface sediment samples were collected using a clamshell grab in November 2019, including 3 from the cut-flow sections and 21 from polluted sections evenly distributed along the river. The locations of all sampling sites were determined using the global positioning system and are shown in Figure 1. Collected from the middle of the riverbed at a depth of 0.2 m, the sediment samples comprising a mixture of minerals, clay and organic matter were stored in clean zip-bags and then transferred to the laboratory. After air-drying, stones and plant materials were removed from the sediment samples which subsequently were ground and passed through a 1-mm nylon screen in preparation for both physical and chemical property analysis. In addition, an agate mortar and pestle were used to grind a quarter of the total samples, which were then filtered through a 0.149-mm nylon screen for measuring the heavy metals.

Following collection and preparation, the samples were analyzed as follows: Sediment pH was determined using a pH meter. OM and TN were determined by means of the potassium dichromate and Kjeldahl methods, respectively. After the samples were digested by HClO_4_-H_2_SO_4_, the ammonium molybdate spectrophotometric method was used to measure TP. Once the samples were whole digested using the HNO_3_-HClO_4_-HF method, the concentrations of Cd, Cr, Cu, Ni, Pb and Zn were measured using an inductively coupled plasma atomic emission spectrometer (ICP-AES, Thermos Scientific, Waltham, MA, USA; detection limit: Cu = 0.54, Ni = 0.28, P = 4.97 μg/L). When the soil samples were digested with HNO_3_: HCl = 1:3, we used atomic fluorescence spectrometry (AFS, AFS-3100, Skyray Instrument, China; detection limit: As, Pb, Hg = 0.01, Cd = 0.001, Zn = 1.0 μg/L) to analyze the concentrations of As and Hg. Furthermore, the national standard soil reference material (GSS-2), repeat analyses and blank analyses were incorporated into the analytical process to test the accuracy of the measurement results. The results show that the analytical error of parallel samples was less than 5%, and the recovery rate of each element was between 91–108%, which was consistent with quality control requirements.

### 2.3. Assessment of Contaminations in Sediment

Assessments of contamination in the sediment samples were carried out using two well-known indexes, namely, the Geo-accumulation index, and the Potential ecological risk index. Their descriptions and particular application in the study are as follows.

#### 2.3.1. Geo-Accumulation Index

The geo-accumulation index (*I*_geo_) is a quantitative index as defined by Müller [26] and is widely used to assess heavy metal pollution in sediments of rivers and lakes [27,28,29]. The equation for calculating *I*_geo_ is as follows:

(1) $$I_{\mathrm{geo}}=log_{2}\left[C_{i}/\left(k\times B_{i}\right)\right]$$

where $C_{i}$ is the measured concentration of element *i* in the sediment, and $B_{i}$ is the geo-chemical background value of element i. In this study, soil in Kaifeng has obvious texture levels and the parent material of the soil is alluvial material from the Yellow River. The soil type is mainly yellow fluvo-aquic soil, hence, the background value for the Chinese fluvo-aquic soil element is adopted in this study [30]. The background values of heavy metals in soil in this study are as follows: Hg = 0.032, Cd = 0.09, As = 9.30, Cu = 22.90, Pb = 20.60, Ni = 28.10, Cr = 64.81, Zn = 67.80. k is a constant (and generally takes on a value of 1.5) to minimize the effect of possible variations in the crustal contribution to sediments [31]. The *I*_geo_ is categorized into seven classes, as shown in Table 1.

#### 2.3.2. Potential Ecological Risk Index (*RI*)

From the sedimental perspective, the *RI* proposed by Hakanson [16] was used to evaluate the heavy metal pollution in the sediments according to heavy metal properties and environmental behavior characteristics. The concentration of each heavy metal, the toxicity level, environmental effect and comprehensive pollution degree of the heavy metals were all considered. The equations for the calculations are as follows:

(2) $$E_{r}^{i}=T_{r}^{i}\times \frac{C_{s}^{i}}{C_{n}^{i}}$$

(3) $$RI=\sum_{i=1}^{n}E_{r}^{i}=\sum_{i=1}^{n}T_{r}^{i}\times C_{f}^{i}=\sum_{i=1}^{n}T_{r}^{i}\times \frac{C_{s}^{i}}{C_{n}^{i}}$$

where $E_{r}^{i}$ is the potential ecological risk factor of the heavy metal i, $C_{s}^{i}$ is the measured concentration of metal i, $C_{n}^{i}$ is the geo-chemical background value for metal i, as adopted from the fluvo-aquic soil background values in China [30], $C_{f}^{i}$ is the contamination factor of the heavy metal, $T_{r}^{i}$ is the toxic effect factor for metal i, as adopted from the relevant study [21], and its values are Hg = 40, Cd = 30, As = 10, Cu = Pb = Ni = 5, Cr = 2, Zn = 1, and n is the number of metals at the site. *RI* is the index of comprehensive potential ecological risk. It can be seen from Equations (2) and (3) that the classification of *RI* should be related to the types and quantities of pollutants involved in the assessment, in which the type affects the toxic effect factor for each metal and the quantity determines the total value of *RI*. This paper referred to the original Hakanson classification system, took its unit toxicity coefficient (see details following), and re-adjusted $E_{r}^{i}$ and *RI* according to the eight heavy metals of pollutants that were evaluated in this paper, so as to judge the ecological risk of water bodies more accurately [17,20]. The $E_{r}^{i}$ classification’s upper limit of the first risk level was obtained by multiplying the non-polluting pollution coefficient ($C_{f}^{i}$= 1) along with the maximum toxicity coefficient of the pollutant under evaluation; in addition, the upper limit values of other levels can be obtained by multiplying the upper limit of its previous level by two. Among the eight heavy metals in this study, the $T_{r}^{i}$ value of Hg was the largest (40), and was used as the first class’s upper limit. Therefore, the classification standard of $E_{r}^{i}$ was obtained. Then the *RI* classification standard was re-adjusted as follows: first, according to Hakanson’s first upper limit value (150), we divided by the sum value of the toxicity coefficients of eight pollutants (133); thereby, the *RI* classification value of unit toxicity coefficient (1.13) was obtained. This value was then multiplied by the sum value of the toxicity coefficients of the eight heavy metals in this study (98), followed by rounding to the nearest integer of tens place (1.13 × 98 = 110.74 ≈ 110) to obtain the first-order classification value. Finally, the classification value of other levels was obtained by multiplying the first-order classification value by two (Table 2).

## 3. Results and Discussion

### 3.1. Physicochemical Properties and Heavy Metal Concentrations in the Sediments from the Huafei River

**Figure 2:**
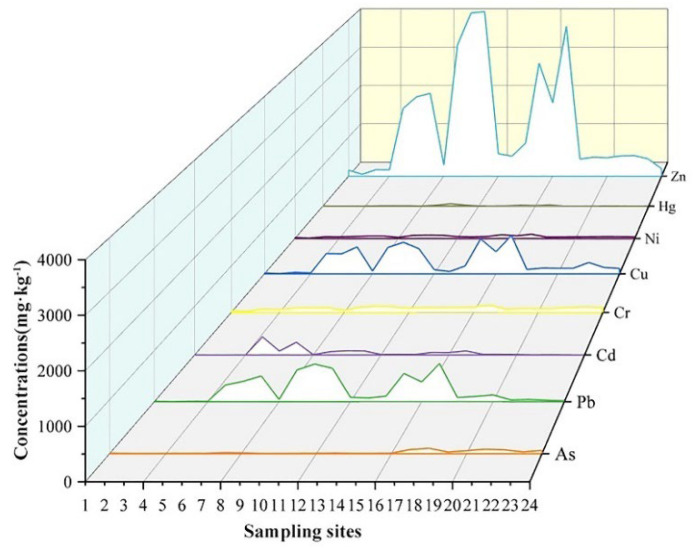
The concentrations of heavy metals in the sediments from the Huafei River.

The result show that the pH of the surface sediments from the Huafei River range from 7.31 to 8.23, and the concentrations of TN, OM and TP range from 587.65 to 5401.84 mg·kg^−^^1^, 472.63 to 13122.10 mg·kg^−^^1^ and 344.81 to 1496.07 mg·kg^−^^1^, respectively, with an average concentration of 2089.76 mg·kg^−^^1^, 5932.59 mg·kg^−^^1^ and 786.58 mg·kg^−^^1^ (Table 3). The descriptive statistics of heavy metal concentrations in topsoil of the Huafei River are described in Table 4. The average concentrations of As, Cd, Cr, Cu, Hg, Ni, Pb and Zn were 26.62, 50.76, 97.08, 292.37, 10.01, 50.13, 238.59 and 1335.12 mg·kg^−^^1^, respectively, which were 2.86, 563.99, 1.50, 12.77, 312.82, 1.78, 11.58 and 19.69 times the background value of fluvo-aquic soil [30]. As shown in Figure 2, the most serious pollution with Cd, Cr, Cu, Hg, Ni, Pb and Zn was found at sites 5–7, 9–10 and 15–17, which were located in the midstream and downstream sections of the river. The highest concentration of As occurred in sites 17–24, which were located in the downstream section of the river.

The coefficients of variation (CV) of Cd, Hg, As, Pb, Zn and Cu were high, measuring 176%, 137%, 113%, 110%, 104% and 94%, respectively (Table 3), indicating that the spatial differences of heavy metal concentrations in the river sediment are large, and the accumulation of these heavy metals in river sediment has been significantly affected by human activities. 

### 3.2. Pollution and Risk Assessment of Heavy Metals in the Sediments

#### 3.2.1. *I*_geo_

**Figure 3:**
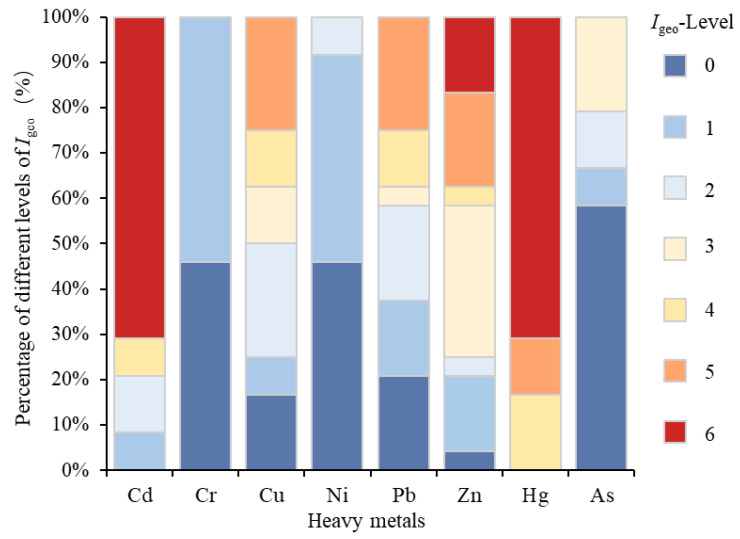
Geo-accumulation indexes level distribution of heavy metals in sediments from the Huafei River. Note: 0: Unpolluted. 1: From unpolluted to moderately polluted. 2: Moderately polluted. 3: From moderately to strongly polluted. 4: Strongly polluted. 5: From strongly to extremely polluted. 6: Extremely polluted.

The geo-accumulation index level distribution for eight heavy metals found in the sediments of the Huafei River are shown in Figure 3. The sequence of the average *I*_geo_ from high to low is Hg (6.36) > Cd (6.12) > Zn (2.81) > Cu (2.19) > Pb (1.61) > Ni (0.11) > As (0.07) > Cr (−0.10). Both Hg and Cd exhibited as extremely polluted (class 6), the highest pollution level, and about 71% of the sampling sites are polluted with this classification. Contaminations with Zn and Cu exhibited as moderately to strongly polluted (class 3), and about 21% and 25% of the sampling sites fluctuated from strongly to extremely polluted by Zn and Cu (class 5), respectively. Contaminations with Pb are classed as moderately polluted, with about 13% and 25% of the sampling sites being strongly polluted and strongly–extremely polluted, respectively. In contrast, As pollution is relatively light (the mean value of the *I*_geo_ is 0.07), and the *I*_geo_ class of Cr and Ni in the Huafei River is 0 and 1, respectively, suggesting that most sections of the river remain relatively clean. The sequence of average *I*_geo_ also indicates that the heavy metals Hg and Cd contribute the most to the geo-accumulation index.

#### 3.2.2. *RI*

The sequence of the average $E_{r}^{i}$ values for the eight heavy metals from high to low are Cd (16919.71) > Hg (12512.80) > Cu (63.84) > Pb (57.91) > As (28.62) > Zn (19.69) > Ni (8.92) > Cr (3.00) (Table 5). Overall, Hg and Cd are at very high ecological risk levels, and all sampling sites identifying Hg and 79% of sampling sites registering Cd show a very high ecological risk. The average $E_{r}^{i}$ value of Cu and Pb is at a moderate risk level, of which 58% of the sampling sites of Cu and 62.5% of the sampling sites of Pb are at a low ecological risk level, while sites 15 and 17 registered Cu and sites 10 and 17 recorded a high ecological risk level. The $E_{r}^{i}$ mean values of the other four heavy metal elements (As, Cr, Ni and Zn) are at a low ecological risk level, as is the case with more than 75% of the sampling sites, including the levels of Ni and Cr at all sampling sites (Table 6).

The comprehensive potential ecological risk index (*RI*) of the study area ranges from 715.30 to 133,338.38, with an average value of 29,614.48, indicating that the study area is critically in a very high ecological risk level (Table 5). In summary, the results of the potential ecological risk assessments indicate that pollution control should be carried out in the midstream and downstream sections of the river, with a focus on targeting Hg and Cd. 

### 3.3. Pollution Variation Characteristics of Heavy Metals in Sediments

**Figure 4:**
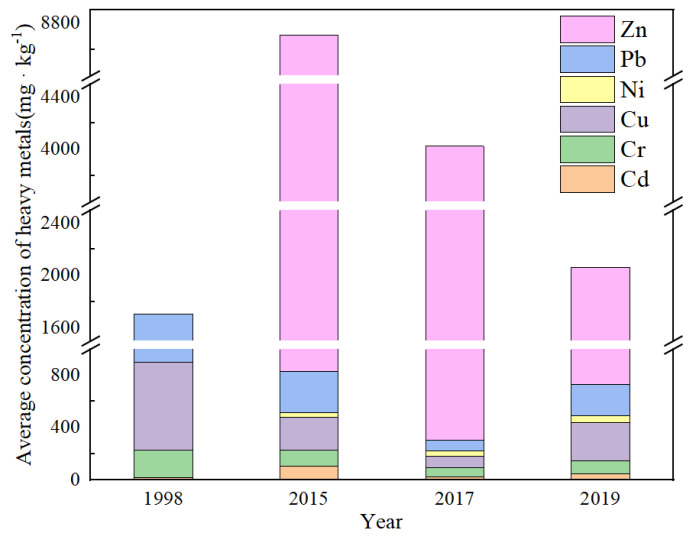
Change in heavy metal concentrations in the sediment from the Huafei River from 1998 to 2019.

**Figure 5:**
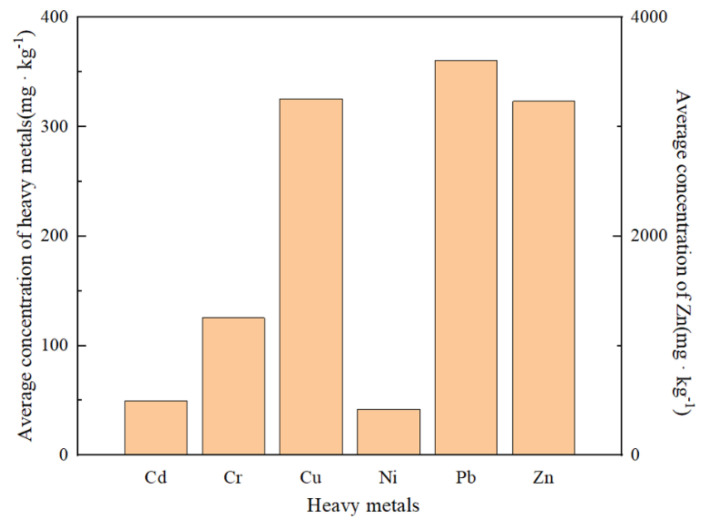
The average concentration of heavy metals over the last 20 years.

The change characteristics of the concentration of heavy metals in the Huafei River sediment over a long-time scale are shown in Figure 4. Figure 4 displays heavy metal data (used in this paper) from studies conducted in 1998 [20], 2015 [25], 2017 [14] and 2019. As can be seen in Figure 4, the concentrations of Cu and Pb decreased significantly in the period 1998 to 2017, and the concentration of Zn also shows a continuous decreasing trend. These decreases can be attributed to the relocation of industrial enterprises from the old industrial base of Kaifeng, the transformation and upgrading of enterprises, and the renewal of equipment. However, the concentrations of some heavy metals (Cu and Pb) increased in 2019, which may be attributed to the fact that the sampling sites in 2019 were mostly located in the midstream and downstream sections of the river. Additionally, with regard to the six heavy metals, the order of their mean concentrations for nearly 20 years followed a general trend of Zn > Pb > Cu > Cr > Cd > Ni, as can be seen in Figure 5, which also indicates that Zn, Pb and Cu dominate the heavy metal pollution of the Huafei River. As compared to other heavy metals, the average concentration of Zn in sediment was found to be higher at more than 3000 mg·kg^−1^, followed by Pb and Cu, with both reaching concentrations of more than 300 mg·kg^−1^.

### 3.4. Identification of Sources of Heavy Metals in Sediments

#### 3.4.1. Correlation Analysis

The results of inter-metal correlations in sediments of the Huafei River using the Pearson correlation method are presented in Table 7, which suggests that Cd has significant positive correlations with Cu and Pb (r = 0.539** and 0.505*, respectively), indicating a common origin. Except for Cd and As, positive correlations are found between the other six elements, with correlation coefficients ranging from 0.627** to 0.985**. Similarly, they may have a common source. The results also suggest that As has no apparent correlations with the other metals. This indicates that As might have different sources compared to the other seven elements.

#### 3.4.2. Principal Component Analysis (PCA)

**Figure 6:**
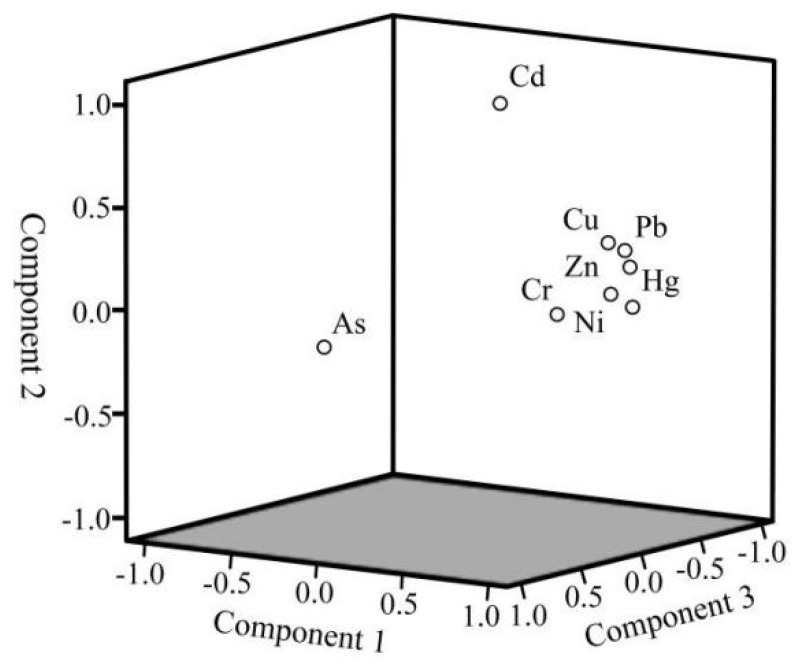
Plot of loading of three principal components in PCA results.

Using varimax rotation with Z-score Standardization, PCA was performed on the heavy metal data, minimizing the sum of the variance of the factor coefficients. This technique divides variables into different groups. In this study, PCA involving the heavy metals was used to further analyze the source of heavy metals. The Keiser–Meyer–Olkin (KMO, 0.793) [32] and Bartlett’s tests (<0.001) [33] indicate that the eight heavy metal concentrations are suitable for PCA. In Table 8, 92.65% of the cumulative variance can be explained through the three principal components (PCs). The rotated component matrix indicates that Cr, Cu, Ni, Pb, Zn and Hg are strongly associated in the PC1 with respective high loadings of 0.827, 0.904, 0.963, 0.920, 0.937 and 0.913. PC2 accounts for 15.66% of the total variance and can be explained through the high loading for Cd (0.965). PC3, dominated by As with a high loading of 0.968, accounts for 14.02%. The spatial scatter diagram of PCA (Figure 6) displays the clustering results of heavy metals more intuitively.

#### 3.4.3. Cluster Analysis

**Figure 7:**
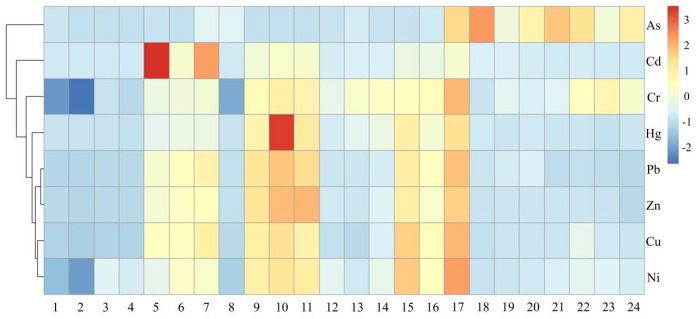
Cluster analysis heat map of heavy metals in sediments from the Huafei River.

Cluster analysis (CA) was applied using Ward’s method and taking the Euclidean distance as a measure, to analyze the source of heavy metals in more significant detail. The CA heat map of heavy metals in river sediments, as shown in Figure 7, suggests the existence of three clusters: (1) comprising Ni, Cu, Zn, Pb, Hg and Cr; (2) Cd; (3) As. Note that the results from CA agree with those derived from PCA.

#### 3.4.4. Identification of Sources of Heavy Metals

According to the results of Pearson’s correlation, principal component and cluster analyses, the sources of heavy metals are divided into three main categories. The first category comprises Cr, Cu, Ni, Pb, Zn and Hg; Cd is in the second category; whilst As is in the third category. In a previous study, industrial activities were reported as playing an important role in contaminations with Cu, Zn and Pb [34]. Similar studies demonstrated that Zn, Cr, Ni, Hg and Cu contaminations were mainly related to industrial activities [35,36]. Moreover, Ji et al. [37] also reported that Pb, Zn and Ni contaminations were associated with industrial production, especially with industrial wastewater from factories. In this research, located in the old industrial zone of Kaifeng, the Huafei River has been heavily affected by industrial activities, although several factories have moved out of the area and, in those remaining, equipment has been updated in recent years. There are many industrial enterprises along the midstream and downstream sections of the river, such as a paper factory, pharmaceutical factory, fertilizer factory, instrument manufacturer, and a metal smelting plant, etc. The industrial wastewater produced by these enterprises is discharged directly into the river without even the simplest form of treatment, whilst fly ash is deposited in the river through the atmosphere, resulting in the accumulation of heavy metals in the river sediments. However, the pollution from Cu and Pb mainly originates from traffic sources [34,35]. In particular, Cu and Pb are released into the environment as a result of the wear and subsequent loss of material from auto parts, as well as through the use of lubricants and petrol [35,38]. Moreover, Kumar et al. [39] concluded that traffic emissions are also one of the sources of Pb, Zn and Ni pollution. The Longhai Railway, the G220 National Highway and the Xincao Road cross the Huafei River, and there are numerous auto-repair plants along the river, all of which contribute to the heavy metal pollution in the river sediments. However, it is suggested that Cr and Ni came from natural origins [31,36,40,41,42,43]. In the present study, the mean concentrations of Cr and Ni are 1.50 and 1.78 times larger than the local background values, respectively; results that are nevertheless not high. Moreover, the CVs of Cr and Ni are in the relatively low spatial variability category, with recorded measurements of 32% and 43%, respectively (Table 4). Therefore, natural sources are another major source for Cr and Ni in the sediments. As mentioned above, the first category is recognized as industrial activities, followed by traffic sources and then natural sources.

In the second category, the only metal element found is Cd, and its accumulation in sediment is reported to stem from industrial activities [34,36,39]. Sewage treatment and the sewage outlets of various plants contribute to Cd contamination [37,44]. Moreover, an additional source of Cd in sediment was found to emanate from agricultural activities, through the intensive use of chemical fertilizers and pesticides [39,42,45]. Furthermore, besides factories, there is a lot of farmland in the vicinity of the river, which potentially contributes to the Cd contamination. Thus, the high concentration of Cd is effectively derived from a combination of industrial and agricultural sources.

In the third category, arsenic (As) is often associated with industrial activities. Industrial sewage, coal combustion and mineral-smelting activities are all related to As pollution in the river sediment [31,36,40]. Therefore, a variety of industrial activities have a significant impact on As pollution.

In summary, the heavy metal contamination is derived from industrial activities, agricultural sources, traffic sources and natural sources, but especially from industrial sources.

### 3.5. Comparison with Those in Other Rivers of the World

Comparisons of the mean values of heavy metal concentrations with those in other regions are listed in Table 9. It was found that As, Cd and Pb had a higher concentration than those reported for the Wen-Rui Tang River by Xia et al. [46] and the Shiqiao River [47] and Buriganga River of Bangladesh [48], while Cr had a lower concentration. The concentrations of Cr, Cu, Ni and Pb in sediments of the Kabini River [49] are about 50 to 2000 times higher than that of the Huafei River of this study. However, all the heavy metal concentrations in sediments of the Huafei River and the above four rivers which are greatly affected by industrial activities are much higher than those from less affected areas, such as the Huangbian River [50], Majia River [50], Liaohe River [40], Hunza River [51] and Brisbane River [5], included in Table 9. Thus, anthropogenic impact, especially industrial activities, is likely to cause serious heavy metal pollution in river sediments.

## 4. Conclusions

In the present study, the pollution characteristic, ecological risk assessment and source identification of eight heavy metals, including As, Cd, Cr, Cu, Hg, Ni, Pb and Zn, in the surface sediments from the Huafei River have been analyzed. The results show that the concentrations of As, Cd, Cr, Cu, Hg, Ni, Pb and Zn are over 2.86, 563.99, 1.50, 12.77, 312.82, 1.78, 11.58 and 19.69 times higher than the local background value, respectively. The geo-accumulation index (*I*_geo_) and potential ecological risk evaluation (*RI*) indicated an extreme pollution level of heavy metals in the sediments. The midstream and downstream sections of the Huafei River are strongly polluted. Hg and Cd are the main pollution factors of the surface sediments of the Huafei River, while Cu, Cr, Zn, As, Ni and Pb had low ecological risks. Long-time scale trend analysis results show the concentrations of Cu and Pb decreased significantly from 1998 to 2017, and then increased in 2019; however, the concentration of Zn showed a continuous decreasing trend. Correlation, PCA and cluster analysis indicated that Cr, Cu, Ni, Pb, Zn and Hg contaminants were mainly derived from industrial activities, traffic sources and natural sources; Cd mainly originated from industrial and agricultural activities; and As sources were mainly associated with industrial activities. In summary, heavy metal pollution in Huafei River surface sediments requires great attention. Important strategies and strong ecological governance should be implemented to reduce discharge of industrial wastewater within the Huafei River. Furthermore, controlling anthropogenic emissions of pollution and conducting long-term monitoring of higher contentions of heavy metals (Hg and Cd) is required to decrease ecological risks from Hg and Cd pollution.

## Figures and Tables

**Table 1:** The geo-accumulation index classification standard.

The Geo-Accumulation Index (*I*_geo_)	Class	Pollution Level
*I*_geo_ ≤ 0	0	Unpolluted
0 < *I*_geo_ ≤ 1	1	From unpolluted to moderately polluted
1 < *I*_geo_ ≤ 2	2	Moderately polluted
2 < *I*_geo_ ≤ 3	3	From moderately to strongly polluted
3 < *I*_geo_ ≤ 4	4	Strongly polluted
4 < *I*_geo_ ≤ 5	5	From strongly to extremely polluted
*I*_geo_ > 5	6	Extremely polluted

**Table 2:** The adjusted $E_{r}^{i}$ and *RI* classification standards.

$E_{r}^{i}\;\mathrm{Value}$	Ecological Risk Levels of a Single Metal	*RI* Value	Ecological Risk Levels to the Environment
<40	Low ecological risk	<110	Low ecological risk
40~80	Moderate ecological risk	110~220	Moderate ecological risk
80~160	Considerable ecological risk	220~440	Considerable ecological risk
160~320	High ecological risk	≥440	Very high ecological risk
≥320	Very high ecological risk		

**Table 3:** Descriptive statistics of physical and chemical properties of the surface sediments from the Huafei River.

	pH	TN (mg·kg^−1^)	TP (mg·kg^−1^)	OM (mg·kg^−1^)
Average	7.73	2089.76	786.58	5932.59
Maximum	8.23	5401.84	1496.07	13,122.10
Minimum	7.31	587.65	344.81	472.63

**Table 4:** Descriptive statistics of heavy metal concentrations (mg·kg^−1^).

	As	Cd	Cr	Cu	Hg	Ni	Pb	Zn
Average	26.62	50.76	97.08	292.37	10.01	50.13	238.59	1335.12
Median	9.55	11.72	102.05	137.23	3.20	43.61	95.32	523.95
Maximum	100.04	371.47	160.97	866.90	56.92	101.35	745.76	4206.97
Minimum	4.91	0.15	23.13	7.70	0.40	11.33	2.80	50.00
SD	30.18	89.11	31.23	275.65	13.68	21.51	262.88	1394.02
CV(%)	113	176	32	94	137	43	110	104
Background of Chinese fluvo-aquic soil	9.30	0.09	64.81	22.90	0.032	28.10	20.60	67.80

**Table 5:** The potential ecological risk indexes of heavy metals in sediments from the Huafei River.

Samping Points	Cd	Cr	Cu	Ni	Pb	Zn	Hg	As	*RI*	*RI* Level
1	83.42	1.22	4.31	3.81	2.02	2.24	652.60	9.72	759.34	Very high
2	163.88	0.71	1.68	2.02	0.68	0.74	1060.19	5.73	1235.63	Very high
3	50.47	2.36	7.34	7.41	2.49	2.46	698.61	5.66	776.81	Very high
4	150.05	2.20	4.46	7.05	2.41	2.38	541.47	5.29	715.30	Very high
5	123,823.82	2.96	99.71	8.50	79.91	25.53	9290.63	7.34	133,338.38	Very high
6	26,556.22	3.11	98.52	10.63	97.04	29.96	12,124.13	5.69	38,925.30	Very high
7	87,100.00	3.19	132.64	10.52	122.45	31.24	12,199.62	20.86	99,620.51	Very high
8	1545.36	1.42	13.61	4.91	10.53	4.21	1485.98	18.67	3084.70	Very high
9	22,482.55	3.72	130.39	13.08	150.52	49.50	28,908.59	5.65	51,744.02	Very high
10	28,851.26	4.11	155.27	14.34	178.78	61.56	71,146.54	5.28	100,417.14	Very high
11	27,645.11	3.91	124.38	13.47	158.43	62.05	33,870.61	5.69	61,883.64	Very high
12	3213.45	2.88	19.91	7.96	21.12	8.46	4638.47	7.33	7919.60	Very high
13	4395.60	3.36	11.48	6.88	18.08	7.57	7937.35	13.04	12,393.36	Very high
14	3416.67	3.49	38.96	8.45	26.54	12.42	12,375.63	6.17	15,888.32	Very high
15	17,298.11	3.55	172.19	15.95	134.31	42.64	31,768.86	6.69	49,442.29	Very high
16	16,201.86	3.65	108.95	11.33	94.01	27.67	17,757.24	10.82	34,215.53	Very high
17	27,816.55	4.97	189.28	18.03	181.01	56.57	38,051.43	82.91	66,400.76	Very high
18	4719.18	2.35	21.34	6.35	20.27	6.44	3356.16	107.57	8239.66	Very high
19	4427.86	2.72	28.54	6.65	25.15	7.18	2574.30	31.76	7104.16	Very high
20	3154.05	2.61	27.52	6.92	32.49	6.98	2723.45	59.18	6013.20	Very high
21	482.37	2.69	27.43	7.29	8.65	7.68	2400.77	90.72	3027.60	Very high
22	1979.37	3.55	55.97	7.92	10.54	7.78	1875.00	76.45	4016.58	Very high
23	416.25	3.84	31.39	7.60	7.57	6.54	493.95	35.10	1002.23	Very high
24	99.60	3.33	26.77	7.00	4.87	2.80	2375.63	63.58	2583.57	Very high
average	16,919.71	3.00	63.84	8.92	57.91	19.69	12,512.80	28.62	29,614.48	Very high

**Table 6:** The amount of each heavy metal at each ecological level.

	Cd	Cr	Cu	Ni	Pb	Zn	Hg	As
Low		24	14	24	15	19		18
Moderate	1		1		1	5		3
Considerable	3		7		6			3
High	1		2		2			
Very high	19						24	

**Table 7:** The correlation coefficients between heavy metals in sediments of the Huafei River.

Pearson Correlation	Cd	Cr	Cu	Ni	Pb	Zn	Hg	As
Cd	1							
Cr	0.242	1						
Cu	0.539 **	0.719 **	1					
Ni	0.332	0.837 **	0.934 **	1				
Pb	0.505 *	0.670 **	0.961 **	0.910 **	1			
Zn	0.446 *	0.689 **	0.933 **	0.907 **	0.985 **	1		
Hg	0.267	0.627 **	0.819 **	0.819 **	0.883 **	0.908 **	1	
As	−0.204	0.136	−0.093	−0.046	−0.181	−0.188	−0.197	1

Note: * At the 0.05 significance level; ** At the 0.01 significance level.

**Table 8:** Rotated component matrix of factor loading.

Elements	1	2	3
Cd	0.221	**0.965**	−0.098
Cr	**0.827**	0.062	0.294
Cu	**0.904**	0.368	−0.022
Ni	**0.963**	0.134	0.042
Pb	**0.920**	0.313	−0.140
Zn	**0.937**	0.239	−0.153
Hg	**0.913**	0.029	−0.209
As	−0.050	−0.095	**0.968**
Eigenvalue (total)	5.038	1.253	1.122
% of total variance	62.974	15.659	14.020
% of cumulative	62.974	78.633	92.653

**Table 9:** Heavy metal concentrations in sediment samples from the Huafei River and other selected rivers from the references (mg·kg^−1^).

	As	Cd	Cr	Cu	Hg	Ni	Pb	Zn	Reference
Huafei River, China	26.62	50.76	97.08	292.37	10.01	50.13	238.59	1335.12	This study
Wen-Rui Tang River, China		17.7	193	310			115	1362	Xia et al. [46] (2018)
Shiqiao River, China		2.79	133	100		66	96	327	Xiao et al. [47] (2013)
Buriganga River, Bangladesh	19.25	7.29	1399	61.86		50.00	68.36	54.54	Bhuiyan et al. [48] (2015)
Kabini River, India			254,520	110,550		91,120	11,670		Hejabi et al. [49] (2011)
Huangbian River, China	7.49	0.35	46.46	30.09		22.71	24.12	90.30	Yang [50] (2017)
Majia River, China	12.58	0.33	64.85	26.21		21.10	20.69	114.05	Yang [50] (2017)
Liaohe River, China	9.88	1.20	35.06	17.82		17.73	10.57	50.24	Ke et al. [40] (2017)
Hunza River, Pakistan		1.11	62.3	36.4		52.6	14.9	54.3	Kashif et al. [51] (2020)
Brisbane River, Australia	3.9	0.3	15	29	0.4	15.3	25.6	106.6	Duodu et al. [5] (2017)

## Data Availability

Data are contained within the article.
